# Editorial: Recent advances in the treatment of epilepsy

**DOI:** 10.3389/fphar.2024.1444138

**Published:** 2024-07-02

**Authors:** Khojasteh Malekmohammad, Antonella Riva, Mahmoud Rafieian-Kopaei

**Affiliations:** ^1^ Department of Biology, College of Sciences, Shiraz University, Shiraz, Iran; ^2^ IRCCS Istituto Giannina Gaslini Institute, Genoa, Italy; ^3^ Department of Neurosciences Rehabilitation, Ophthalmology, Genetics, Maternal and Child Health (DiNOGMI), University of Genoa, Genoa, Italy; ^4^ Medical Plants Research Center, Basic Health Sciences Institute, Shahrekord University of Medical Sciences, Shahrekord, Iran

**Keywords:** epilepsy, seizure, drug-resistance, anti-seizure medications, ketogenic diet, neuroprotection

Epilepsy is one of the World’s oldest recognized neurological disorders, while its intricate mechanisms of pathogenesis, development, and treatment remain a conundrum. This Research Topic aimed to delve into the recent advancements in the treatment of epilepsy. It compiles unique studies from original research to review articles, covering the pathophysiological mechanisms underlying epilepsy with a special focus on the novel pharmacological and non-pharmacological therapeutic approaches.

Anti-seizure medications (ASMs) are the preferred first line of treatment to reduce neuronal excitability and seizure frequency. Various drugs, including brivaracetam, cannabidiol, cenobamate (CNB), everolimus (EVE), and fenfluramine have been employed in patients with epilepsy (Schmidt and Schachter, 2014; Perucca, 2021). Despite the wide usage of ASMs, about 30% of patients with epilepsy are refractory to treatment. Drug-Resistant Epilepsy (DRE) is a condition with long-term negative outcomes due to the unsatisfactory control over seizures (Gonzalez-Giraldo and Sullivan, 2020; Löscher et al., 2020).

Pietrafusa et al. reported that CNB, a drug acting both as a positive GABA_A_ allosteric modulator and as an inhibitor of persistent sodium currents, could reduce the frequency of seizures in patients with drug-resistant focal-onset epilepsy. In their work the Authors provided pharmacokinetic evidence of drug-drug interactions and assessed CNB tolerability and efficacy. In line with these findings, Park et al. found that EVE, an inhibitor of the mammalian target of rapamycin complex 1 (mTORC1), could be a valuable adjunctive treatment for patients with seizures associated with focal cortical dysplasia.

Phenobarbital (PB) and levetiracetam (LEV) are generally the first choice for treating neonatal onset seizures. According to the intriguing research work of Quinlan et al. PB can cause long-term/adverse effects on the developing brain based on the transcriptomic analysis. Impaired synaptic development, behavioral and cognitive changes, and acute neurotoxicity are the most common manifestations of PB. LEV is safer than PB; it can prevent these changes in the brain. Mamad et al. research highlighted the anti-seizure effects of P2X7 receptor antagonist JNJ-47965567 in the treatment of drug-resistant Temporal Lobe Epilepsy (TLE). The JNJ-47965567 mechanisms of action in reducing spontaneous recurrent seizures can be based on decreasing astrogliosis, therefore reducing neuroinflammation and changing the morphology of microglia processes in the CA3 region of the hippocampus. Research by Bastaki et al. revealed the anticonvulsant effect of histamine H3 receptor antagonist DL76 against maximal electroshock (MES)-induced seizure. Also, DL76, used at high doses, did not show urogenital and skeletal abnormalities and caudal malformation.

In line with the use of natural compound-based treatments, *Lippa origanoides* essential oil (LOEO) demonstrated to control seizures induced by pentylenetetrazol in rats. Also, *L. origanoides* essential oil had a synergistic anticonvulsant effect if associated to diazepam. A combination of LOEO with diazepam may be considered in the future as a neuroprotective therapeutic option for the treatment of epilepsy (Bastos de Araújo et al.).

Benzyl isothiocyanate (BITC), a natural component of cruciferous vegetables, exhibited its neuroprotective properties by improving the cognitive function, learning and memory abilities, and spatial cognition in the lithium-pilocarpine-induced chronic temporal lobe epileptic mice. BITC treatment increased the antioxidant capacity of the hippocampal tissue through activating of nuclear factor E2-related factor2/heme oxygenase 1 (Nrf2/HO-1) signaling pathway, along with increased glutathione peroxidase (GSH-Px) activity and decreased malondialdehyde (MDA) content (Xiaoyu et al.).

Zoungrana et al. reviewed the mechanisms involved in the induction, development, and pathogenesis of epilepsy. Also, they highlighted the Activated Protein C (APC) neuroprotective effects and the possible connection between APC and epileptic seizures. APC, as an anticoagulant protein, can reduce epilepsy pathogenesis by preventing blood-brain barrier dysfunction, decreasing neuroinflammation and apoptosis, and eventually increasing neurogenesis.

Freidel et al. shed light on the effects of different psychedelics, especially classical psychedelics, on non-epilepsy chronic seizure disorders. In their review paper, they provided a complete background about Psychedelic-assisted therapy (PAT) in the context of epilepsy and seizures. Usage of psychedelics such as Ketamine, lysergic acid diethylamide (LSD), 3,4 methylenedioxymethamphetamine (MDMA), and psilocybin “magic mushrooms” may be safe in patients with epilepsy under clinical supervision.

Drug resistance is a main challenge in the treatment of epilepsy. It is often related to uncontrolled tonic-colonic seizures that can result in sudden unexpected death (SUDEP). Neurostimulation techniques have gained more attention as novel non-pharmacological treatments for DRE patients. Particularly, Vagal Nerve Stimulation (VNS), Deep Brain Stimulation (DBS), and Responsive NeuroStimulation (RNS) are invasive techniques to modulate neuronal activity (Davis and Gaitanis, 2020; Riva et al., 2021). Also, there are 4 types of non-invasive neuromodulation for the treatment of epilepsy: transcutaneous vagus nerve stimulation (tVNS), trigeminal nerve stimulation (TNS), repetitive transcranial magnetic stimulation (rTMS), and Focused ultrasound method (Riva et al., 2021; Lescrauwaet et al., 2022).

The Ketogenic Diet (KD), a dietary approach with low-carbohydrate and high-fat content, has a pivotal role in the treatment of DRE along with surgery, ASMs, and neuromodulatory techniques. The KD and its variants, including the Classic Ketogenic Diet (KD), Medium-Chain Triglyceride Diet (MCTD), Modified Atkins Diet (MAD), and Low-Glycemic Index Treatment (LGIT) have already proven effective in seizure reduction through different anti-seizure mechanisms, such as elevating inhibitory neurotransmitters and reducing neuronal excitability, increasing brain energy production, and reducing production of proinflammatory and pro-apoptotic factors (Verrotti et al., 2020; Haridas and Kossoff, 2022).

The gut microbiota is also related to epilepsy through the gut-brain axis. Genetic factors, age, region, and diet are various factors that can affect the gut microbiota composition. The KD has been widely used in the treatment of refractory epilepsy. The antiepileptic effects of the KD on the gut microbiota have been studied through 16S rRNA sequencing in patients with mitochondrial epilepsy (Wang et al.).

Furthermore, the review by Zhu et al. marked a close relationship between the microbiota and the gut-brain by elucidating how the rebuilding of gut microbiota by KD, probiotics, and fecal microbiota transplantation could improve drug-resistant epilepsy. Indeed, the mechanisms through which the gut microbiota can be modulated for the treatment of epilepsy have been described.

Building upon the understanding of the molecular mechanisms of the KD in the treatment of epilepsy, in their study, Lin et al. investigated the mechanistic role of KD in treating epilepsy by assessing the Adenylate Cyclase 3 (ADCY3)-initiated cAMP signaling pathway. It was revealed that KD could improve epileptic seizures by regulating fatty acid metabolism, activating ADCY3-initiated cAMP signaling pathway, and enhancing neuronal inhibition. Also, Meta-analysis and animal experiments proved that KD was superior to other diets (i.e., routine diet) in contrast in treating epilepsy.

Therefore, the study of mechanisms underlying epilepsy and its novel treatments has become an intriguing scientific field. We hope that this special edition will present new insights into the current knowledge about ASMs and non-pharmacological therapeutic options involved in the treatment of epilepsy. We anticipate that the articles published on this Research Topic would be useful for clinicians and researchers in the field of neuroscience.
